# Progress in Otology

**Published:** 1900-06-09

**Authors:** 


### Progress in Otology.

(*Continued from page* 155.)

**The Mastoid Operation in Chronic Otorrhœa.**—The Royal Medical and Chirurgical Society has devoted two meetings to a discussion on this subject.[6-7] The following remarks have been abstracted from the reports of the proceedings. C. A. Ballance alluded to the difficulty in the selection of cases for the operation. There was a risk in delay on the one hand; on the other, of the performance of an unnecesary operation. He proposed that typical intractable cases should be treated by two operations; first, the complete mastoid operation for the cure of the disease; and, secondly, the operation for healing the wound by the epithelial grafting of the raw bone cavity. Notes of 20 cases were appended. Sir W. Dalby read a paper on the cases suitable for the mastoid operation. The cases in which the operation was undoubtedly necessary were: Firstly, cases in which septicæmia had commenced; secondly, cases in which there was carious bone in the tympanic cavity accompanied by ominous symptoms; and, thirdly, whenever there was evidence of mastoid disease of long or short standing. The question was open when, though there was carious bone present, there were no ominous symptoms, or when, without bone disease or ominous symptoms, there was intractable otorrhœa. Cases occurred, also, in which a less complete operation was necessary. A large number of persons went through long lives with perforations which at times discharged slightly, at other times became dry, and many of them heard well and had little inconvenience, and it was a very serious question whether this class should be submitted to the extensive operation. A. E. Cumberbatch considered the complete operation of exposing the antrum and throwing the iter, attic, and tympanum into one cavity was usually inexpedient in the following groups of cases: Firstly, where the greater part of the membrane was destroyed, and the mucous lining of the tympanum was polypoid, but without obvious bone disease. Secondly, where there was frequent recurrence of the discharge on slight provocation, though it was readily arrested again. In these the perforation of the membrana remained permanent, or closed when the discharge ceased. Thirdly, where there was discharge from both ears, but in which hearing was excellent, and there were no symptoms calling for operation beyond the discharge. On the other hand, there were four groups of cases in which the operation should be performed: (1) In those recurrent cases in which the onset of discharge was always associated with malaise, headache, and rise of temperature, with occasional mastoid discomfort; (2) "spoilt ears," which, having given no trouble for many years, suddenly developed symptoms of labyrinthine vertigo, pointing either to spread of inflammation to the labyrinth, or to some accumulation causing pressure in that region; (3) cases of intermittent discharge, where inspection revealed masses of soddened epidermis, often hiding small granulations, and where syringing constantly removed white shreddy patches, and when the usual treatment failed to cure; (4) cases of periodic attacks of mastoid pain, commencing after all signs of active mischief in the ear had ceased, suggesting the existence of scleorosing osteitis. He considered Ballance's operation to be nearly perfect. W. Milligan considered Ballance's technique constituted a distinct advance. The period of after-treatment was thereby much shortened. In an ordinary Stacke's operation it took four months for complete cuticularisation, and here it could be completed in three or four weeks. Dundas Grant said pain and vertigo were very strong indications for operation, unless explained by visible and removable causes. Sometimes vertigo ceased after the removal of a granulation which was pressing on the stapes. Caries was also a decided indication, and the occurrence of facial paralysis or of nerve deafness, also the failure of general health. The degree of hearing afforded an indication. If a whisper could be heard at three feet distance the tympanic apparatus was still of some value. If the hearing power were below that the operation might be the more readily agreed to. The responsibility in deciding against operation was greater than in deciding for it, owing to the danger of the supervention of meningitis. Percy Jakins[6] records a consecutive series of 80 cases of Stacke's radical mastoid operation. Broadly speaking the operation consists in laying open the mastoid antrum and any diseased mastoid cells connected with it, opening the aditus leading to the attic of the tympanum, cutting away all overhanging bone, removing the posterior wall of the osseous metus, and in some cases removing the malleus and incus. In several of the cases it was necessary to lay bare the dura mater in the middle fossa by removing necrosed bone. Sometimes the groove of the lateral sinus was exposed. It was only occasionally that the mastoid cells became involved and required to be dealt with. Audition was seldom rendered worse by the operation, and in some was materially improved. The youngest patient was 14 months old. In two cases facial paralysis appeared after the operation, but in both it disappeared again within nine months. Three of the cases died; in each the patient had been brought into the hospital in a very advanced state of pyæmic poisoning. The mortality of the operation, except in such cases, is practically nil. In all cases, in time, the antrum attic and whole tympanum become soundly healed.

[6] Lancet, Jan., 1900. [7] Lancet., Feb., 1900.

